# Identification of wild soybean miRNAs and their target genes responsive to aluminum stress

**DOI:** 10.1186/1471-2229-12-182

**Published:** 2012-10-05

**Authors:** Qiao-Ying Zeng, Cun-Yi Yang, Qi-Bin Ma, Xiu-Ping Li, Wen-Wen Dong, Hai Nian

**Affiliations:** 1 State Key Laboratory for Conservation and Utilization of Subtropical Agro-bioresources, College of Agriculture, South China Agricultural University, Guangzhou, 510642, China

**Keywords:** Wild soybean, Aluminum stress, miRNA, High-throughput sequencing

## Abstract

**Background:**

MicroRNAs (miRNAs) play important regulatory roles in development and stress response in plants. Wild soybean (*Glycine soja*) has undergone long-term natural selection and may have evolved special mechanisms to survive stress conditions as a result. However, little information about miRNAs especially miRNAs responsive to aluminum (Al) stress is available in wild soybean.

**Results:**

Two small RNA libraries and two degradome libraries were constructed from the roots of Al-treated and Al-free *G. soja* seedlings. For miRNA identification, a total of 7,287,655 and 7,035,914 clean reads in Al-treated and Al-free small RNAs libraries, respectively, were generated, and 97 known miRNAs and 31 novel miRNAs were identified. In addition, 49 p3 or p5 strands of known miRNAs were found. Among all the identified miRNAs, the expressions of 30 miRNAs were responsive to Al stress. Through degradome sequencing, 86 genes were identified as targets of the known miRNAs and five genes were found to be the targets of the novel miRNAs obtained in this study. Gene ontology (GO) annotations of target transcripts indicated that 52 target genes cleaved by conserved miRNA families might play roles in the regulation of transcription. Additionally, some genes, such as those for the auxin response factor (ARF), domain-containing disease resistance protein (NB-ARC), leucine-rich repeat and toll/interleukin-1 receptor-like protein (LRR-TIR) domain protein, cation transporting ATPase, Myb transcription factors, and the no apical meristem (NAM) protein, that are known to be responsive to stress, were found to be cleaved under Al stress conditions.

**Conclusions:**

A number of miRNAs and their targets were detected in wild soybean. Some of them that were responsive to biotic and abiotic stresses were regulated by Al stress. These findings provide valuable information to understand the function of miRNAs in Al tolerance.

## Background

Soybean (*Glycine max*) is one of the most widely grown crop species in the world. Current evidence indicates that the cultivated soybean was domesticated from its annual wild relative, wild soybean (*Glycine soja* Sieb. and Zucc*.*), over 5,000 years ago in China [1]. Compared to cultivated soybean, wild soybean possesses much higher adaptability to natural environmental stresses such as drought, alkaline and salt stress, which demonstrates the potential usefulness of the wild soybean to improve cultivated soybean [2-5]. Soybean breeding and improvement is hindered by a narrow domesticated germplasm compared to other crop species [6]. Studies have revealed that *G. soja* shows greater genetic diversity and higher allelic diversity than *G. max*[6-8]. Wild soybean can readily cross with cultivated soybean giving rise to fertile hybrids, thus making *G. soja* a promising source of novel genes and alleles for soybean breeding and improvement.

Aluminum (Al) toxicity is a major limitation to crop production on the acid soils that make up about 30–40% of the world’s arable lands. In acid soil, aluminum causes the rapid inhibition of root growth and subsequently inhibits water and nutrient uptake in plants, which increases the susceptibility of plants to environmental stresses and results in reductions in crop production [9,10]. Soybean is planted widely on acid soil and its productivity is significantly hampered by Al stress. It is known that plant species and genotypes within species differ markedly in their tolerance to excess Al; however, the mechanisms responsible for Al tolerance are not so clearly understood. The exclusion of Al from the roots and the detoxification of Al ions in the plant are two of the Al tolerance mechanisms that have been proposed in plants [11]. To date, many of the genes responsible for Al tolerance that have been identified in plants other than soybean are involved in root Al-induced organic acid exudation, the redistribution or sequestration of Al, and in coding transcription factors [12-18]. In soybean, Al tolerance has been described as a quantitative trait that involves several genes and pathways [19,20]. Ermolayev and coworkers [21] and Ragland and Soliman [22] have identified some genes that were related to Al tolerance in soybean. These genes include phosphoenolpyruvate carboxylase (PEPC), homolog of translationally controlled tumor proteins (TCTPs), inosine 5^′^-monophosphate dehydrogenases (IMPDHs) [21], aluminum-induced 3–2 (*Sali3-2*), and aluminum-induced 5-4a (*Sali 4-5a*) [22]. Duressa and coworkers [23] used DNA microarray technology to identify putative genes in the Al-tolerant soybean line PI 416937 and reported that many genes involved in transcription activation, stress response, cell metabolism and signaling were differentially expressed in Al-tolerant soybean. They concluded that Cys2His2 and ADR6 transcription activators, cell wall modifying enzymes, and phytosulfokine growth factors might play roles in soybean Al tolerance [23].

MicroRNAs (miRNA), one of the major types of endogenous non-coding RNAs in higher plants, modulate gene expression at the post-transcription and translational levels [24,25]. An increasing amount of evidence demonstrates that miRNAs play critical roles in regulating development, stress response, hormone response and many other biological processes in plants [26-28]. Although a number of miRNAs have been identified in plants, the genome-wide discovery of new miRNAs is essential for the functional characterization of miRNAs. Recently, using next-generation sequencing technology, hundreds of small RNAs, especially miRNAs with low abundance, have been isolated by small RNA sequencing in higher plants [29-32]. Further, this technology has been used successfully to systematically identify stress-associated miRNAs [33-37]. Currently miRBase (Release 18: November 2011) lists 362 miRNAs that have been identified in *G. max* from different tissues, including root, seed, flower, nodule and shoot apical meristem [38-43]*.* Recently, miRNAs responsive to abiotic and biotic stresses such as water deficit, rust and *Phytophthora* root rot have also been reported in soybean [44,45]. However, in the same release of miRBase 18.0, only 13 miRNAs from wild soybean are listed [46].

To functionally characterize the biological roles of each miRNAs, target validation is required. Modified 5’ RACE (rapid amplification of cDNA ends) has been widely applied in target confirmation and cleavage site mapping [26]. However, this method is used only for small-scale target confirmation because it is costly, and labor and time consuming. Recently, degradome sequencing, a high-throughput method known as PARE (parallel analysis of RNA ends), has been adopted to identify the target transcripts of miRNAs [47-49]. This technology has been successfully used to identify hundreds of targets cleaved by conserved and unconserved miRNAs in plant species [36,47,49-51].

Most of the soil in South China where wild soybean is widely distributed is typical acidic soil. Therefore, the wild soybean that grows there may have evolved special mechanisms to survive under Al stress conditions. However, little information about the response of wild soybean to Al stress is available. To obtain highly Al tolerant plant materials, the core germplasm of more than 500 wild soybean plants from South China were collected and screened. From among the 500 plants, one wild genotype (BW69) showed the highest Al tolerance (unpublished). Subsequently, this genotype was treated with Al to detect new miRNAs and their targets responsive to Al stress in wild soybean. The microRNA sequencing and degradome sequencing developed by Solexa (Illumina Inc.) were applied to investigate the expression of miRNAs and their targets responsive to Al stress. In total, we identified 97 known miRNAs, 31 novel miRNAs, and a further 49 p3 or p5 strands of known miRNAs. In addition, 91 genes sliced by miRNAs were detected through degradome sequencing. Among the cleavage targets, 52 genes were transcription regulators.

## Results

### Deep-sequencing results of wild soybean

**Figure 1:**
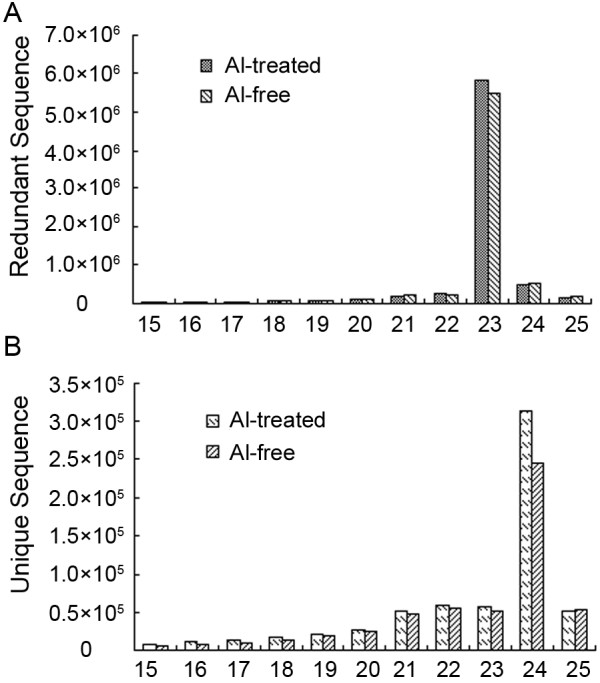
**Length distribution of the wild soybean samll RNAs obtained by high-throughput sequencing in two libraries.A**) Size distribution of redundant sequences. **B**) Size distribution of unique sequences.

To identify miRNAs responsive to Al stress, two small RNA libraries constructed from the roots of Al-treated and Al-free (the control) wild soybean seedlings were sequenced on the Illumina Genome Analyzer IIx. A total of 8,616,284 and 8,712,410 raw sequences were generated from Al-treated and Al-free libraries, respectively (Table 1) (high-throughput sequencing data were deposited into the NCBI-GEO with accession no. GSE38065). After removal of low-quality and corrupted adapter sequences (such as 3’ adapter not found), sequences with less than 15 bases after cutting 3’ adapter, and junk reads, 7,287,655 and 7,035,914 clean reads from the Al-treated and Al-free libraries, respectively, remained (Table 1). The length distribution of the unique sequences showed that the most abundant sequences were 24 nt long; however, when redundant sequences were included, the most abundant sequences were 23 nt long (Figure 1). This atypical situation was also reported in cucumber in which a high number of redundant 22-nt sequences were obtained by Solexa (Illumina) high-throughput sequencing [52,53]. When the clean reads were searched against the Rfam [54] and repeat databases [55], 2.68% and 2.37% of the small RNAs in the Al-treated and Al-free libraries, respectively, were removed from the libraries (Table 1). The remaining sequences were mapped to the *G.max* genome sequence [56], and sequences that aligned to mRNAs were removed. Finally, 782,582 and 754,685 sequences in the Al-treated and Al-free libraries, respectively, were obtained and used for miRNA prediction (Table 1).

**Table 1:** **Distribution of the *G. soja *sequences in the Al-treated and Al-free libraries**

**RNA class**	**Al-treated**		**Al-free**	
	**Counts**	**Percentage of total**	**Counts**	**Percentage of total**
Raw reads	8,616,284	100.00%	8,712,410	100.00%
Number of reads removed due to 3ADT^a^ not found	974,498	11.31%	1,411,300	16.20%
Number of reads removed due to <15 bases after 3ADT cut	345,537	4.01%	260,266	2.99%
Junk reads	8,594	0.10%	4,930	0.06%
Number of mapped reads	7,287,655	84.58%	7,035,914	80.76%
mRNA^b^	6,310,043	86.59%	6,114,595	86.91%
Rfam^c^	192,054	2.64%	164,494	2.34%
Repeats^d^	2,976	0.04%	2,140	0.03%
Known and predicted miRNAs	184,734	2.53%	124,343	1.77%
No hit	597,848	8.20%	630,342	8.96%

a, 3ADT is the 3’ adaptor.

b, The *G. max* mRNAs were downloaded from ftp://ftp.jgi-psf.org/pub/JGI_data/phytozome/v7.0/Gmax/annotation/Gmax_109_transcript.fa.gz[56].

c, Rfam (V 10.0) was downloaded from ftp://ftp.sanger.ac.uk/pub/databases/Rfam/9.1/[54].

d, Repbase (V13.12) was downloaded from http://www.girinst.org/repbase/update/index.html[55].

The total number of small RNAs for miRNA prediction in the Al-treated library (782,582) was 3.69% higher than in the Al-free library (754,685), and the total number of known and predicted miRNA sequences in the Al-treated library (184,734) was 1.49-fold higher than in the Al-free library (124,343) (Table 1). A total of 177 miRNAs were identified in this study. Among them, 92.10% of the miRNAs detected in the Al-treated library were also found in the Al-free library, whereas, 3.95% of the miRNAs were found only in the Al-treated library, and 3.95% miRNAs were found only in the Al-free library (Additional file 1, Table 2).

**Table 2:** Identification of novel microRNAs in wild soybean

**miR_name**^**a**^	**Sequenced sequence**	**Length**	**genome ID**	**strand**	**dG**	**CG%**	**MFEI**	**raw_Al-free**	**raw_Al-treated**	***Glycine max *Genome**^**b**^	***Glycine soja *Genome**^**c**^
PC^d^-1-5p	TGGCCTGTGATGCATCAATTTGTC	24	Gm12	+	−26.6	44	0.7	49	10	yes	no
PC-1-3p	GTCTCTGATGAATGCTCAAATTCT	24	Gm12	+	−26.6	44	0.7	7	11	yes	no
PC-4-5p	GTCGTTGTAGTATAGTGGTAAGT	23	Gm05	+	−33.5	37.3	0.7	26	8	yes	yes
PC-4-3p	GTCGTTGTAGTATAGTGGTAAGTA	24	Gm05	+	−33.5	37.3	0.7	84	48	yes	yes
PC-8-5p	ATTGTTGAGACGATTCTGTAGACG	24	Gm09	+	−16.8	28.3	0.6	12	9	yes	no
PC-12-5p	AAATCTACATGCGGATCAAGTTGA	24	Gm17	+	−34.2	39.7	1.1	0	12	yes	yes
PC-20-3p	CAACGGTCATAATGTAGATTTA	22	Gm05	+	−29	40.6	0.7	10	16	yes	yes
PC-21-5p	CAAAGATTCATCGTAGGCTAGACT	24	Gm08	+	−25.1	55.4	0.7	14	12	yes	yes
PC-25-3p	CTACATAAGGCACGAGATCATC	22	Gm18	-	−17	38.2	0.7	7	29	yes	yes
PC-26-3p	CATCGGTCGAGAGCGTTCTT	20	Gm06	-	−48.8	46.2	0.9	14	6	yes	no
PC-28-3p	CATTTGGGCACCTATTTTGACTC	23	Gm06	+	−18.8	40	0.8	24	2	yes	yes
PC-32-3p	TATATTCGGATATTCACATT	20	Gm02	+	−26.7	34.3	0.8	15	10	yes	no
PC-33-3p	GGAGAACAAAGAAGCAGCTAAATTC	25	Gm16	+	−22.4	32.6	0.7	4	29	yes	no
PC-35-5p	AAAAGGACACATGACTCACACCTA	24	Gm10	-	−11.9	31.2	0.5	10	10	yes	yes
PC-36-5p	GAGTTGGCGAGTTGGACACGTGGC	24	Gm08	-	−38	59.5	0.8	9	12	yes	no
PC-39-5p	TAGATTTTAAAGTTGCGGATCA	22	Gm04	-	−42.3	38.5	1.1	10	32	yes	yes
PC-40-3p	AATACGTAAGGCTTGAGCTTGACT	24	Gm01	-	−37.8	42.9	1	8	17	yes	yes
PC-41-3p	AAATCAGATGATATGGACTTAAAT	24	Gm13	-	−21.2	28.3	0.8	19	20	yes	yes
PC-42-5p	AAGAAACGTTGACTCTCCGTGTT	23	Gm03	-	−39.9	38.5	0.8	11	5	yes	no
PC-43-5p	ATCGGGATGCTCAGTTCGCATGGT	24	Gm10	+	−33.9	46.6	1	5	11	yes	yes
PC-44-3p	ATGAACCCTTTGAGATCACTGGTT	24	Gm06	-	−26.2	47.1	0.7	17	13	yes	yes
PC-45-5p	AGACGGTAAGAAGAGAATTTCAAT	24	Gm01	-	−14.3	23.8	0.8	11	4	yes	yes
PC-46-5p	CTATATGATGAAGATA	16	Gm18	+	−28.3	34	0.9	52	26	yes	yes
PC-47-3p	CGTTGTAGTATAGTGGTAAGTATT	24	Gm19	+	−31.1	46	0.5	9	11	yes	yes
PC-48-3p	TTAGCTTCTTTCACCTTTCCC	21	Gm17	-	−41.8	41.5	1.2	135	137	yes	no
PC-52-5p	TGAGGGCAAAGATATTAGAGAA	22	Gm05	-	−26	35.9	0.7	5	12	yes	yes
PC-56-3p	GGCGAGGAATCTGGGCTCATT	21	Gm15	+	−37.5	47.6	1	16	19	yes	yes
PC-57-3p	GCATACAGGGAGTCAAGCAGA	21	Gm09	-	−48	43	1.2	20	27	yes	no
PC-58-5p	TTAGTTGAATGGTACTGTAGTAGT	24	Gm06	+	−29.2	37	0.7	6	11	yes	yes
PC-60-3p	TTTCCCGGCAATGGAACCA	19	Gm17	+	−19.3	38	0.5	298	208	yes	no
PC-61-5p	AACTTACTGACTCGTTGACTCGGT	24	Gm14	+	−21.1	39.4	0.8	10	5	yes	yes

Genomic locations of the pre-miRNA are based on the *Glycine max* assembly Gmax_109_repeatmasked.fa downloaded from ftp.plantgdb.org/download/Genomes/GmGDB/. Identification of known miRNAs was based on miRBase 18.0.

a, miR_name is the name that was assigned to the detected miRNA sequences.

b, *G. max* Genome is the precursor sequences whether they could be mapped to Glycine max [56].

c, *G. soja* Genome is the precursor sequences whether they could be mapped to Glycine soja EST or GSS [92].

d, PC means 'predicted candidate'.

### Identification of known miRNAs in wild soybean

To identify known miRNAs in the two libraries, the clean reads were compared with known miRNA precursor or mature miRNA sequences in miRBase 18.0 allowing no more than two mismatches. A total of 97 known miRNAs were identified in the two samll RNA libraries (Additional file 1). Based on their similarity to the known miRNAs, the *G. soja* miRNAs were classified into two groups. Group I comprised 57 unconserved miRNAs that were either present in wild soybean or other legumes (Additional file 1). Thirteen wild soybean specific miRNAs were described in miRbase 18.0 [46] and all but one of them (gso-miR3522b) were identified in our libraries. Most of these wild soybean specific miRNAs were relatively highly expressed in the two root libraries; among them, gso-miR2218 was the most abundant in the two libraries (7,282 and 19,061 reads in the Al-free and Al-treated libraries, respectively), followed by gso-miRNA1509a. However, with the exception of PN-miR1509b (PN indicates a potentially novel miRNA), most of the legume-specific miRNAs had relatively lower levels of expression. The largest miRNA family in the two libraries was PN-miR1520 with 4 members. Group II contained 40 highly conserved miRNAs (Additional file 1). Of these, the most abundant was miR159a with 2,363 and 2,121 reads in the Al-free and Al-treated libraries respectively. Conserved miRNAs are known to have important functions in plant development and stress response [26]. In the present study, 19 highly conserved miRNA families were identified. The largest conserved miRNA family was miR156 with 9 members.

The p3/p5 strands of known miRNAs have been used as strong evidence to identify miRNAs [57] and 49 p3/p5 strands of known miRNAs were detected in the present study (Additional file 1). Generally, p3/p5 strands of miRNAs are thought to be more unstable than other miRNAs in cells, and the numbers of them have been assumed to be ten times less than those of other mature miRNAs [26,58]. In the present study, the p3/p5 strands of most of the known miRNAs had relatively low expression. However, the PN-mir4415-p3, gso-mir2109-p3 and gso-mir1510b-p5 sequences occurred more frequently than the corresponding mature miRNAs, suggesting that these candidates might be true mature miRNAs.

### Identification of novel miRNAs from wild soybean

The formation of stable hairpin structures has been suggested as a prerequisite for the annotation of new miRNAs [57,59]. To identify novel miRNAs, we used the M-fold web server to predict the secondary structures of the candidate miRNA precursors [60] and found that 29 of the potential pre-miRNAs met this requirement. Two of the 29 pre-miRNA were predicted to generated two miRNAs, one from the 3’ arm (3p) and one from the 5’ arm (5p) (Table 2). The 31 novel miRNAs were 16 to 25 nt long; 51.61% of them were 24 nt long (Table 2). Most of novel miRNAs had relatively low expressions, and only 12 of the novel miRNA candidates had more than 20 reads in the two libraries (Table 2). The novel miRNAs were all generated from one locus.

**Figure 2:**
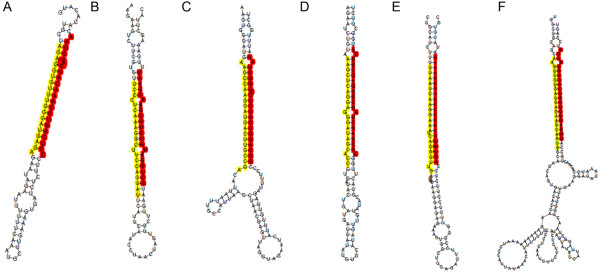
Precursors structures of six pairs of miRNA/miRNA*s strictly meet characteristics mentioned by Meyers *et al.* (**A**-**F**) The precursor sequences of PN-miR1507c/PN-miR1507c*, PN-miR862a/PN-miR862a-5p, PN-miR169c/PN-mir169c-p3, PN-miR390b/PN-mir390b-p3, PN-miR1509b/PN-mir1509b-p3 and PN-miR4415/PN-mir4415-p3, respectively.

When the strict criteria defined by Meyers and coworkers [57] was used to filter all the results, totally six pairs of miRNA/miRNA* strictly met all three of those characteristics. Those miRNAs were PN-miR1507c/PN-miR1507c*, PN-miR862a/PN-miR862a-5p, PN-miR1509b/PN-mir1509b-p3, PN-miR169c/PN-mir169c-p3, PN-miR390b/PN-mir390b-p3 and PN-miR4415/PN-mir4415-p3 (Figure 2). However, because of the unstable of miRNA* in cells [26], when we filtered the results, we did not strictly regard to parameter of miRNA and miRNA* which were derived from opposite stem-arms and formed a duplex with two nucleotide, 3’ overhangs.

### Identification of Al-responsive wild soybean miRNAs

To identify miRNAs responsive to Al stress, the differential expression of miRNAs in the two libraries was compared using the read counts obtained from the high-throughput sequencing. Because some of the miRNAs in this study had extremely low abundances, which might lead to false results, known miRNAs with less than 10 raw reads and novel miRNAs with less than 20 raw reads in the Al-free and Al-treated libraries were removed from the expression analysis. In the two libraries, miRNAs with log_2_ fold changes higher than 1 were designated as ‘up-regulated’. Similarly, miRNAs with the log_2_ fold changes less than −1 were designated as ‘down-regulated’. Thirty miRNAs belonging to 26 families were differentially expressed between the two libraries (Table 3); 12 miRNAs were down-regulated and 18 were up-regulated by Al exposure. Of the 12 wild soybean specific miRNAs, gso-miR2218, the miRNA that had the highest expression abundant in the two libraries, was up-regulated by Al stress. Seven legume-specific miRNAs were response to Al stress. Among them, PN-miR1509b had the highest up-regulated fold change. The p3/p5 strands of five unconserved miRNAs (gso-mir1509a-p3, gso-mir1510a-p5, gso-mir2109-p3, PN-miR4387e-p5 and PN-mir4415-p3) were differentially expressed between the two libraries. Among the highly conserved miRNAs, five (PN-miR169c, PN-miR390b, PN-miR396a and PN-miR403a, b) were up-regulated and five belonging to four conserved families (PN-miR156b,c,d, PN-miR164 and PN-miR2111) were down-regulated in response to Al stress. Seven novel miRNAs showed differential expression between the two libraries; three were up-regulated and four were down-regulated.

**Table 3:** Profiles of the differentially expressed miRNAs responsive to Al stress

**miR**^**a**^**_name**	**Sequenced sequence**	**raw_Al-free**	**raw_Al-treated**	**norm_ Al-free**	**norm_ Al-treated**	**Log**_**2**_**(Al-treated/Al-free)**
PC^c^-28-3p	CATTTGGGCACCTATTTTGACTC	24	2	25.36	1.89	−3.74
PN^b^-miR164	TGGAGAAGCAGGGCACGTGCA	95	12	100.39	11.36	−3.14
PN-miR2111	CTAGTCCTTGGGATGCAGATTACG	57	10	60.23	9.46	−2.67
PC-1-5p	TGGCCTGTGATGCATCAATTTGTC	49	10	51.78	9.46	−2.45
PC-4-5p	GTCGTTGTAGTATAGTGGTAAGT	26	8	27.47	7.57	−1.86
PN-miR5044	GTAGTGGATGCCTAGAGGTCC	25	8	26.42	7.57	−1.80
PN-miR156b,c,d	TGACAGAAGAGAGTGAGCAC	369	137	389.92	129.65	−1.59
gso-mir2109-p3	GGAGGCGTAGATACTCACACC	2655	1201	2805.55	1136.55	−1.30
PC-46-5p	CTATATGATGAAGATA	52	26	54.95	24.60	−1.16
PN-miR1507c	CCTCATTCCAAACATCATCTAA	377	194	398.38	183.59	−1.12
gso-miR2218	TTGCCGATTCCACCCATTCCTA	7282	19061	7694.91	18038.15	1.23
PN-miR169c	AAGCCAAGGATGACTTGCCGA	17	47	17.96	44.48	1.31
PN-miR390b	AAGCTCAGGAGGGATAGCACC	13	36	13.74	34.07	1.31
PN-miR862a, b	GCTGGATGTCTTTGAAGGAAT	113	355	119.41	335.95	1.49
PC-39-5p	TAGATTTTAAAGTTGCGGATCA	10	32	10.57	30.28	1.52
gso-mir1509a-p3	ACCGTGTTTCCTTGGTTAACG	9	34	9.51	32.18	1.76
PC-25-3p	CTACATAAGGCACGAGATCATC	7	29	7.40	27.44	1.89
gso-mir1510a-p5	AGGGATAGGTAAAACAATGAC	6	25	6.34	23.66	1.90
PN-miR4387e-p5	TCACGCCTAATCACTGACGCA	11	49	11.62	46.37	2.00
PN-miR1514a	TTCATTTTTAAAATAGGCATTGGG	11	51	11.62	48.26	2.05
PN-mir4415-p3	TTGATTCTCATCACAACATGG	41	243	43.32	229.96	2.41
PC-33-3p	GGAGAACAAAGAAGCAGCTAAATTC	4	29	4.23	27.44	2.70
PN-miR4369	GGATCAAGCTGATCCGGAAGTGGA	3	23	3.17	21.77	2.78
PN-miR396a-1	TTCCACAGCTTTCTTGAACTG	81	676	85.59	639.72	2.90
PN-miR403a, b	TTAGATTCACGCACAAACTTG	5	44	5.28	41.64	2.98
PN-miR1509b	TTAATCAAGGAAATCACGGTTG	190	2232	200.77	2112.23	3.40

a, miR_name is the name that was assigned to the detected miRNA sequences.

b, PN means ‘potential novel’.

c, PC means 'predicted candidate’.

**Figure 3:**
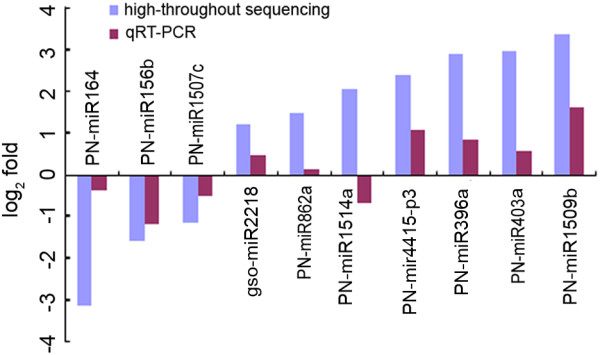
**Differential expressions of ten miRNAs that were responsive to Al stress** .

Based on the high-throughput sequencing results, ten miRNAs were selected for qRT-PCR to validate their expression patterns. As shown in Figure 3, the expression patterns of three of the selected down-regulated miRNAs and five of the selected up-regulated miRNAs obtained by qRT-PCR was similar to results from high-throughput sequencing; however, in the qRT-PCR analysis, the expression of PN-miR862a did not change under Al stress, and the qRT-PCR expression pattern of PN-miR1514a was not consistent with that from the high-throughput sequencing. The fold changes obtained from the qRT-PCR were much lower than that those obtained from the high-throughput sequencing, possibly because of differences in the sensitivity and specificity between qRT-PCR and high-throughput sequencing technology.

### Identification of targets of miRNAs in wild soybean

**Figure 4:**
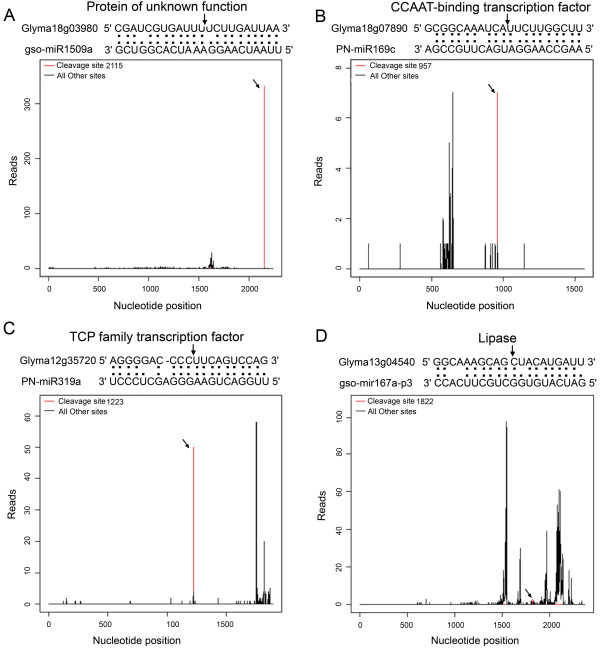
**T-plots of miRNA targets in the four different categories.** The T-plots show the distribution of the degradome tags along the full-length of the target mRNA sequence (bottom). The red line represents the sliced target transcripts and is shown by an arrow. The alignments show the miRNA with a portion of its target sequence (top). Two dots indicate matched RNA base pairs; one dot indicates a GU mismatch. The arrow shows the cleavage site. (**A**) Example of the category I target Glyma18g03980 for gso-miR1509a. (**B**) Example of the category II target Glyma18g07890 for PN-miR169c. (**C**) Example of the category III target Glyma12g35720 for PN-miR319a. (**D**) Example of the category IV target Glyma13g04540 for gso-mir167a-p3. The categories were based on the relative abundance of the tags at the target sites.

Target validation is important to thoroughly elucidate the biological roles of miRNAs. To date, only two targets for wild soybean miRNAs have been identified by 5’RACE [46]. To identify the targets cleaved by the candidate miRNAs identified in the present study, the recently developed high-throughput degradome sequencing technology was adopted to perform a genome-wide analysis of the mRNAs potentially cleaved by the miRNAs [47]. In total, we obtained 16,979,070 and 16,064,291 raw reads from the Al-free and Al-treated libraries, respectively (Additional file 2). After removing the reads without the CAGCAG adaptor, 16,508,834 and 15,539,593 distinct reads in Al-free and Al-treated libraries, respectively, were obtained. The distinct sequences were aligned to the *G. max* genome database, and 8,407,136 and 7,960,260 reads from the Al-free and Al-treated libraries, respectively, were mapped to the genome. The mapped reads from the Al-free and Al-treated libraries represented 50,619 and 51,077 annotated *G. max* genes, respectively (Additional file 2). The CleaveLane pipeline reported previously was adopted to identify the sliced targets for the known miRNAs and novel miRNA candidates [61]. The sliced target transcripts were categorized into four groups according to the relative abundance of the tags at the target mRNA sites (Figure 4).

**Figure 5:**
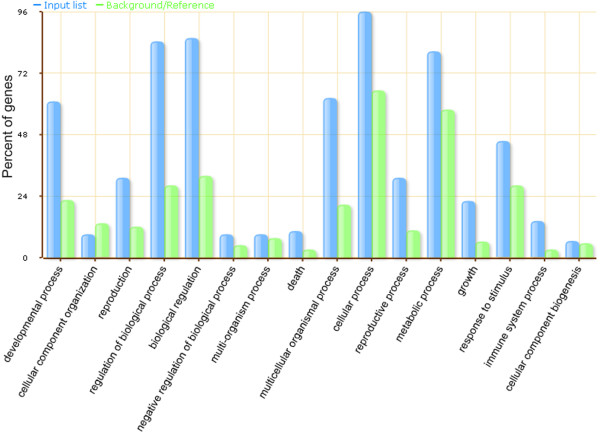
**GO analyses of the targets of the known and new miRNAs in *Glycine soja*.** Blue bars indicate the enrichment of the GO terms in the miRNA targets in GO. Green bars indicate the percentage of the total annotated soybean genes that were mapped to the GO terms.

In total, 91 targets that could potentially be cleaved by the miRNAs were identified in the two libraries (Additional file 3, Table 4). The AgriGO toolkit was used to do gene ontology (GO) analysis [62]. Of the 91 targets, 73 were found to have at least one GO annotation (Figure 5). More than 80% of the target genes were annotated as being involved in regulation of biological processes and this term was more enriched in the miRNA targets than in the soybean genes as a whole. The enrichment of genes involved in the regulation of biological processes may be consistent with the observation that miRNA targets are mainly involved in regulating development [26].

**Table 4:** Identified targets of the novel miRNAs in wild soybean

**miR_name**	**Target**	**Alignment score**	**Cleavage site**	**Al-treated**			**Al-free**			
				**Category**^**a**^	**Percentage of cleavage at the expected site**	**Reads at cleavage site (tpb)**^**b**^	**Category**	**Percentage of cleavage at the expected site**	**Reads at cleavage site (tpb)**	**Target gene family**
PC-46-5p	Glyma03g33240	1	2649	2	0.50%	96.53				Cation transporting ATPase
	Glyma05g23260	1.5	3244				2	0.68%	121.15	Protein tyrosine kinase
	Glyma13g03270	3.5	1559	2	1.33%	38.61	2	2.01%	60.57	TPR repeat-containing protein
	Glyma14g23650	3.5	961	2	1.06%	38.61	2	1.47%	60.57	TPR repeat-containing protein
	Glyma19g35960	1	2649	2	0.51%	96.53				Cation transporting ATPase
gso-mir167a-p3	Glyma13g04540	3.5	1822	3	0.07%	58.99				Lipase (class3)
PN-mir156f-p3	Glyma03g27590	3	333	2	0.17%	96.53	2	0.17%	121.15	Transcription elongation factor SPT6
	Glyma19g30560	3	206	2	0.14%	96.53	2	0.25%	121.15	Transcription elongation factor SPT6

a, The numbers represent the four category as follows: 0 indicates category I; 1 indicates category II; 2 indicates category III; 3 indicates category IV.

b, tpb, transcripts per billion.

In our degradome dataset, 86 target transcripts for 17 known miRNA families were identified in the two libraries (Additional file 3). Of the 86 target transcripts, 34 (39.53%) were found in both libraries, 45 (52.33%) were found only in the Al-treated library, and seven (8.14%) were found only in the Al-free library, showing that miRNA-mediated target cleavage was stimulated by Al stress. Furthermore, among the 86 targets, 16 for five unconserved miRNA families, 66 for 12 conserved miRNA families and three for the two p3/p5 strands of known miRNA were identified (Additional file 3, Table 4). When the transcript abundance and distribution patterns of the targets were analyzed in the two libraries, 23 (29.11%), 50 (63.29%) and six (7.60%) targets in the Al-treated library were found to be distributed into categories I, III and IV, respectively, and eight (19.51%), one (2.44%), 26 (63.41%) and six (14.63%) targets in the Al-free library were grouped into categories I, II, III and IV, respectively (Additional file 3).

**Figure 6:**
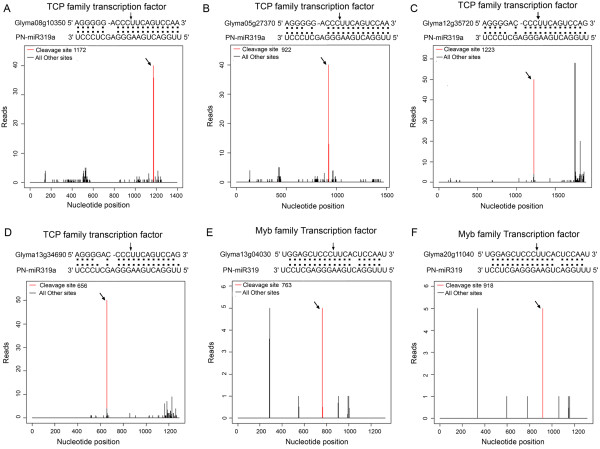
**T-plots of the targets cleaved by the miR319 family.** The T-plots show the distribution of the degradome tags along the full-length of the target mRNA sequence (bottom). The red line represents the sliced target transcripts and is shown by an arrow. The alignment shows the miRNA with a portion of its target sequence (top). The two dots indicate matched RNA base pairs; one dot indicates a GU mismatch. The arrow shows the cleavage site. (**A**, **B**, **C**, and **D**) Examples of different TCP transcription factors as targets for PN-miR319a. (**E**, **F**) Examples of different Myb transcription factors as targets for PN-miR319.

In most cases, the identified miRNAs were predicted to cleave two or more different targets. For example, nine members of CCAAT-binding transcription factor family genes were predicted to be cleaved by PN-miR169 (identified in the Al-treated library), and PN-miR319 was predicted to slice eight genes belonging to the Myb and TCP families of transcription factors (T-plots of six of the targets are presented in Figure 6). In contrast, only one target each was identified for gso-miR1509, gso-miR2109 and PN-miR399 (Additional file 3). Interestingly, some transcripts were found to be regulated by pairs of miRNAs. For instance, PN-miR159 and PN-miR319 targeted two Myb family transcription factors (Glyma13g04030 and Glyma20g11040), and PN-miR156 and PN-miR157 sliced five members of the SBP domain protein family, suggesting that pairs of miRNAs might have a combinatorial function in gene regulation networks (Additional file 3).

The GO annotations of the target transcripts in our study revealed that 52 of the targets that were cleaved by the eight conserved miRNA families played roles in transcription regulation. These miRNA families and their targets are highly conserved in plants, suggesting that the conserved miRNAs might act as master modulators in gene expression networks [26,36]. Of the eight conserved miRNAs, PN-miR169 targeted nine CCAAT-binding transcription factors of which eight were found in both libraries; the other was found only in the Al-treated library (Additional file 3). In contrast, the auxin response factor and Myb transcription factor genes cleaved by miR160, miR159 and miR319, were found only in the Al-treated libraries. However, only three no apical meristem (NAM) protein genes out of the 16 targets cleaved by the unconserved miRNAs, were found to act as transcription factors. All other targets were annotated as involved in different biological functions, suggesting that the unconserved miRNAs might play special roles in gene expression networks.

However, in this study, we found only five unique transcripts belonging to three signal conduct gene families (cation transporting ATPase, protein tyrosine kinase, and TPR repeat-containing protein) cleaved by PC-46-5p. Two TPR repeat-containing proteins were identified in both libraries, two cation transporting ATPases were found only in the Al-treated library and a protein tyrosine kinase was found only in the Al-free library. When the transcript abundance and distribution pattern of the five transcripts were analyzed, all of them fell into category III.

## Discussion

### Identification of wild soybean miRNAs by high-throughput sequencing

Recently, high-throughput sequencing has been used to systemically identify plant miRNAs responsive to abiotic stress in several plant species, and this has greatly advanced our knowledge of the miRNAs functions in stress tolerance [36,37,50]. To study the roles of miRNAs in gene regulation under Al stress, we constructed two libraries from the roots of wild soybean seedlings treated either with Al or without Al. The number of miRNAs identified in our study far exceeds the previous report in which only 15 conserved miRNAs and nine novel miRNAs were identified [46]. Of the miRNAs obtained by high-throughput sequencing, almost half (44.52%) of the known miRNAs had relatively low expression abundance (less than 10 raw reads) (Additional file 1), indicating that high-throughput sequencing is a most powerful strategy for the identification miRNAs, especially those with low expression levels, in plants. When we compared our miRNA dataset to the *G. soja* miRNAs reported previously, we found that most of the known miRNA families had been recovered; however, miR171 and gso-miR3522b were not found in our study (Additional file 1) [46]. This might be because different wild soybean seedling tissues were used in the two studies.

We found that only 10 conserved miRNAs belonging to seven conserved miRNA families were responsive to Al stress. However, 13 unconserved miRNAs and seven novel miRNAs were responsive to Al stress (Table 3). These results indicated that the unconserved miRNAs might play more important roles in the regulation of the plant’s tolerance to Al stress. A previous study of miRNAs in *M. truncatula* using a bioinformatic approach combined with validation by qRT-PCR, found that some conserved miRNAs, such as miR166 and miR398, were down-regulated, and some, miR171, miR319, miR393, and miR519, were up-regulated in response to Al stress [63]. Subsequently, in a high-throughput sequencing study in *M. truncatula*, miR160, miR319, miR396, miR1507 miR1510a and miR390 were found to be down-regulated after treatment with Al, while miR166 and miR171 were not responsive to Al [50]. In this study, miR396 and miR390 were up-regulated in response to Al which are different from the results of Chen and coworkers [50] (Table 3). Furthermore, we found that miR1510a was not responsive to Al stress, while mir1510a-p5 was up-regulated under Al stress. The different results might be caused by differences in the genome and tolerance mechanisms between *M. truncatula* and *G. soja*.

Cross adaptation is a common phenomenon in plants exposed to different combinations of stresses [64]. Researches have revealed that some conserved miRNAs that are responsive to biotic and abiotic stresses might play roles in cross adaptation [50]. In this study, miR156 which were reported earlier to be down-regulated in response to cadmium treatment [65] were found to be down-regulated under Al stress. In Arabidopsis, miR164 was reported to be induced by drought stress which cleaved the NAC1 transcription factor gene leading to the down-regulation of auxin signals and resulting in reduction in lateral root emergence [66]. We found that miR164 was down-regulated under Al stress (Table 3, Figure 3). In plants, miR169 was found to be responsive to abiotic stresses such as cold, drought, and salinity [67-71]. Recently, miR169 were also found to be responsive to some biotic stresses such as a virulent form of the bacterium, *P. syringae pv. tomato* DC3000 in Arabidopsis [72] and *Fusarium virguliforme*, the causal agent of sudden death syndrome, in soybean [73]. Our high-throughput sequencing results showed that miR169 was down-regulated under Al stress (Table 3). These findings suggest that the conserved miRNAs might take part in cross adaptation to regulate plant tolerance to biotic and abiotic stresses.

### The targets of wild soybean miRNAs identified by high-throughput degradome sequencing

In plants, degradome sequencing has been shown to be an efficient strategy to identify targets of miRNAs [47,49,51]. In wild soybean, many miRNA targets have been predicted, but only two of them had been identified by 5^′^-RACE [46]. In this study, 86 target transcripts for 17 known miRNA families and 5 targets for a novel miRNA family were identified through degradome sequencing technology (Additional file 3, Table 4). Previous researches have revealed that miRNAs have a strong preference for genes associated with development, particularly for genes encoding transcription factors and F-box proteins [26]. We found that 52 of the targets for the conserved miRNAs were involved in transcription regulation (Additional file 4); they included the Myb, ARF, WRC, SBP, TCP, SPT6, AP2 and CCAAT type transcription factor gene families which are conserved in other plant species [74-77]. This result suggested that the targets of conserved miRNAs might participate in various aspects of plant development and stress responses and may act as the main nodes in gene expression networks in plants.

In our degradome sequencing experiment, we found 12 conserved miRNA families that had detectable cleavage targets. However, only six unconserved miRNAs and one novel miRNAs had detectable cleavage targets (Additional file 3, Table 4). A similar result was reported in *M. truncatula*[36] and *G. max*[38]. There were two possible explanations for these results. First, in plants, the miRNA regulation mechanism is not only by mRNA cleavage but also by translational repression [24] and second, some targets could not be identified because of differences in the spatial or temporal expression of a miRNA and its target which might cause insufficient degradation of the target [78]. Here, while 16,508,834 and 15,539,593 distinct reads were obtained in the Al-free and Al-treated degradome libraries respectively, only 0.0039% and 0.0065% were identified as targets of miRNAs (Additional file 3, Table 4). The cleavage fragments that were not identified as targets of miRNAs might be caused by random cleavage during gene transcription or by other small interference RNAs.

Moreover, we found that the targets cleaved by miRNAs in the Al-treated library were different from those in the Al-free library. For example, the cleavage fragments of gso-miR2109, PN-miR1514, PN-miR159, PN-miR160, and PN-miR394 were found only in the Al-treated library. Furthermore, cleavage fragment of the eight genes targeted by gso-miR1510 were found in the Al-treated library, but only two of them were identified in the Al-free library (Additional file 3). In our study, the number of genes cleaved by PN-miR1514, PN-miR396 and PN-miR169 which were up-regulated under Al stress was more in the Al-treated library than in Al-free library. In addition, more tags of target transcripts sliced by PN-miR1509, PN-miR393 and PN-miR403 which were up-regulated under Al stress were detected in the Al-free library (Additional file 3, Table 4). This indicated that Al exposure strongly affected the cleavage of target transcripts mediated by miRNAs.

The target transcripts for which cleavage fragments were found only in the Al-treated library were involved in stress responses and included the mRNAs for NB-ARC domain proteins, LRR domain protein, NAM protein, Myb family transcription factors, auxin response factor, and cation transporting ATPase. This finding might be the result of the temporal expression of miRNAs and their target genes. The NB-ARC, LRR and TIR domains have been identified in plant resistance proteins involved in pathogen recognition and subsequent activation of innate immune responses [79-81]. The cleavage of TIR-NBS-LRR type transcripts were reported in *M. truncatula* under mercury stress [36]. We found that NB-ARC domain, LRR domain and TIR domain transcripts (encoding the three domain TIR-NBS-LRR protein) were cleaved by the gso-miR1510 family of miRNAs. Interestingly, the cleavage fragments of NB-ARC domain, LRR protein were found only in the Al-treated library (Additional file 3), suggesting that the TIR-NBS-LRR protein might take part in a regulating pathway involved in the recognition of biotic and abiotic stresses. The cleavage of the TIR-NBS-LRR protein under Al stress might result in the disruption of the immune system which might increase the susceptibility of the plant to other stresses.

The perception and transmission of stress signals mediated by hormones could play important roles in Al tolerance in plants. Recent studies revealed that the inhibition of root elongation, a typical symptom of Al toxicity, might be was caused by disruption of auxin distribution in roots [82,83]. It was reported that cleavage of the auxin response factor (ARF) by miR160 regulated the development of the root cap. In Arabidopsis, when miR160 was over-expressed, three ARF genes were barely detectable and the root length of the transgenic seedlings was reduced [84]. In the present study, seven genes belonging to the ARF family were found to be cleaved by miR160 only in the Al-treated library (Additional file 3). Previously, NAC1 was found to mediate auxin signaling to promote lateral root development [85,86]. Transgenic plants expressing antisense NAC1 cDNA show a reduction of lateral roots. The no apical meristem protein is a member of the NAC domain superfamily. In the Al-treated library, no apical meristem was detected to be cleaved by PN-miR1514 (Additional file 3) and the expression of PN-miR1514 was found to be up-regulated under Al stress. These results suggest that the cleavage of ARF transcripts by miR160 and NAM transcripts by miR1514 might be involved in the plant’s response to auxin which may regulate the inhibition of root development under Al toxicity. In *G. max*, ABA accumulation was induced by Al treatment, and the tendency of ABA to be distributed in the Al-exposed root was shown by a split-root experiment [87]. Research revealed that the Myb transcription factors might take part in the response to ABA accumulation, and the cleavage of Myb transcription factors mediated by miR159 has been reported in Arabidopsis under drought stress [88]. We also found that the cleavage of Myb transcription factors was mediated by miR159 in *G. soja* in this study (Additional file 3). Together these results indicated that the cleavage of hormone-related genes might affect the response of wild soybean to hormones potentially affecting the plant’s tolerance to Al stress and causing metabolic dysfunction.

Peroxide stress often occurs concurrently with Al stress. It has been shown that activated antioxidative enzymes and other antioxidant metabolites are beneficial for plant growth under Al stress, because they may contribute to the removal of excess reactive oxygen species and inhibit lipid peroxidation [89]. Cation transporting ATPase has been reported to plays significant roles in adaptive responses to oxidative stress by removing excessive Ca^2+^ from the cytosol [90,91]. The cleavage of the transcripts of the cation transporting ATPase by PC-46-5p might play a role in antioxidative systems induced by Al toxicity (Additional file 3).

## Conclusions

In our study, two samll RNA libraries and two degradome libraries were constructed from the roots of wild soybean seedlings for deep sequencing. We obtained a total of 8,616,284 and 8,712,410 raw sequences from the Al-treated and Al-free libraries, respectively, and predicted 31 new miRNAs in wild soybean by bioinformatic analysis. We discovered 30 miRNAs that were responsive to Al^3+^. These findings provided valuable information for the identification of miRNAs in wild soybean and could be used for the functional characterization of miRNAs in the response of legume plants to Al^3+^ phytotoxicity. Through degradome sequencing, we detected 91 targets cleavage by conserved, unconserved and novel miRNAs in wild soybean. Some of miRNAs and their targets were related to biotic and abiotic stresses. The expressions of the miRNAs and targets identified in our study were shown to be regulated by Al stress. This finding suggests that Al can trigger protective mechanisms involving miRNAs that can improve the plant’s tolerance to Al toxicity. The identification of the new candidate miRNAs and their target genes should contribute to our understanding of gene regulatory frameworks in plants, and may provide insights into the role of miRNAs and their targets in regulating plant tolerance to Al stress.

## Methods

### Plant culture and treatment

Wild soybean seeds (BW69) were grown in a chamber with the following settings: 70% relative humidity, 28°C / 25°C and a light regime of 14 h light / 10 h dark. Before sowing, episperm of seeds were cut carefully using a knife. The seed surface was sterilized with 70% ethanol for 30 s and subsequently washed in deionized water. Then, seeds were sown in quartz sand and left for 4 d to germinate. Germinated seedlings were then transferred into growth boxes. Sixty seedlings were cultured in a 6 L vessel containing a nutrient solution made up of 0.75 mM KNO_3_, 0.25 mM Ca(NO_3_)_2_.4 H_2_O, 0.325 mM MgSO_4_.7 H_2_O, 20 μM Fe(III)-EDTA, 8 μM H_3_BO_3_, 10 μM KH_2_PO_4_, 0.2 μM (NH_4_)_6_MO_7_O_2_.4 H_2_O, 0.2 μM CuSO_4_.5 H_2_O, 0.2 μM ZnSO_4_.7 H_2_O and 0.2 μM MnCl_2_.4 H_2_O. Two days after transplanting, the seedlings were transferred into nutrient solution with either 0 (Al-free) or 50 (Al-treated) μM AlCl_3_ (pH 4.5). Roots 10 cm from the root apex were harvested at 1, 3, 6, 12, 24, 48 and 96 h after the initiation of Al stress. The samples were immediately frozen in liquid nitrogen, and stored at −80°C. To minimize biological variance, 20 roots from 4 repeats were pooled.

### Small RNA library construction, sequencing and sequencing data analysis

Two sets of sample were prepared; one set was derived from the Al-treated roots harvested at 1, 3, 6, 12, 24, 48 and 96 h time points and the other set was from the Al-free roots harvested at the same time points. Total RNA was isolated with the Total RNA Purification Kit (Norgen Biotek Corporation, Thorold, Canada) according to the manufacturer’s instructions. Small RNA libraries were generated from the two samples using the Illumina Truseq Small RNA Preparation kit (Illumina, San Diego, USA) according to Illumina’s TruSeq Small RNA Sample Preparation Guide. The purified cDNA library was used for cluster generation on Illumina’s Cluster Station and then sequenced on an Illumina GAIIx (Illumina) following the vendor’s instructions for running the instrument. Raw sequencing reads were obtained using Illumina’s Sequencing Control Studio software version 2.8 (SCS v2.8) (Illumina) following real-time sequencing image analysis and base-calling by Illumina's Real-Time Analysis version 1.8.70 (RTA v1.8.70) (Illumina). A proprietary pipeline script, ACGT101-miR v4.2 (LC Sciences, Houston, TX, USA), was used for sequencing data analysis (Li *et al.* 2010; Wei *et al.* 2011). Because of the limited sequence information for wild soybean, the *G. max* database was employed for identification of the miRNAs and for the prediction of secondary structure [56]. Then we blasted all our predicted precursors to 18,511 ESTs and 180153 GSSs of *G. soja* registered in ncbi [92], and we gave an instruction of all the precursor sequences whether they could be mapped to *G. soja* EST or GSS.

### Degradome library construction and bioinformatics analysis

The wild soybean root degradome library was constructed following the methods described previously by German and coworkers [48]. The *G. max* database and transcript sequences were used as the reference sequences [56]. The publicly available CleaveLand 3.0 software package and the ACGT301-DGEv1.0 program (LC Sciences, Houston, TX, USA) were used to detect potentially sliced targets of the known and novel miRNA identified by high-throughput sequencing and degradome analysis [47,61]. The *G. soja* miRNA to *G. max* sequence alignments were then scored as follow: mismatched pairs or single nucleotide bulges were each scored as 1 and GU pairs were scored as 0.5. The mismatched and GU pair scores within the core segment (at positions 2–13) were doubled. All targets were classified into four categories based on the abundance of the resulting mRNA tag relative to the overall profile of degradome reads that matched the target [47,61]. In category I, the abundance of cleavage sequences was equal to the most abundant degradome sequences on the transcript, and there was only one maximum on the transcript; in category II, the abundance of the degradome sequences at the cleavage site was equal to the maximum abundance on the transcript, and there was more than one maximum on the transcript; in category III, the abundance of cleavage sequences was less than the maximum but higher than the median for the transcript; in category IV, the abundance at cleavage site was equal to or less than the median for the transcripts. The optimized score thresholds were set to 4.5 for category I, 4 for category II, 3.5 for category III, and 3 for category IV. These thresholds were used to select the resulting output. The gene ontology (GO) analysis of the candidate target transcripts of the known and new miRNAs identified in this study was performed using the AgriGO toolkit [62].

### Quantitative real-time PCR

Total RNA was extracted from the *G. soja* roots from Al-free and Al-treated samples with TRIZOL reagent following the manufacturer’s instructions (Invitrogen). These samples were collected at the same time as those for miRNAs and degradome sequencing. The total RNA (1 μg) was treated with DNase I and reverse-transcribed using miRNA specific stemloop primers (Additional file 5), reverse-transcriptase and deoxynucleotide triphosphates. qRT-PCR analysis was carried out using the SsoFast EvaGreen Supermix kit (BIO-RAD) in a CXF96 (BIO-RAD) qRT-PCR System with 166 ng cDNA and 6 pmol of each gene-specific primer (Additional file 5). The analysis was performed using two independent cDNA preparations and triplicate PCR reactions. The relative expression ratio was calculated using the 2^-ΔΔCt^ method with ACT3 as the reference gene.

## Competing interests

The authors declare that they have no competing interests.

## Authors’ contributions

ZQY participated in the study design, carried out the material preparation, miRNA sequencing and degradome sequencing data analysis, participated in the qRT-PCR experiments, and drafted the manuscript. YCY participated in the design of the study and assisted with miRNA sequencing, degradome sequencing data analysis, and participated in writing the manuscript. MQB and LXP participated in the design of the study. DWW participated in the qRT-PCR experiments. NH participated in the study design and coordination, and helped with the manuscript editing. All authors read and approved the final manuscript.

## Supplementary Material

Additional file 1 **Identification of known miRNAs obtained by high-throughput sequencing.** Detailed information of total known miRNAs identified in this study. Click here for file

Additional file 2 **Summary of data in the degradome library.** Detailed information of the raw data obtained from degradome sequencing and the distribution of the sequences. Click here for file

Additional file 3: **Targets mRNAs of the known miRNAs in wild soybean.** Detailed information of the targets cleaved by the known miRNAs in this study. Click here for file

Additional file 4 **The distribution of the transcription regulators cleaved by known miRNAs.** The percentage indicates proportion of the different transcription regulators out of the total number of transcription regulators for the known miRNAs. CCAAT, CCAAT-binding transcription factor; Myb, Myb family of transcription factors; SBP, SBP domain proteins; TCP, TCP family of transcription factors; SPT6, transcription elongation factor SPT6; WRC, growth regulating factor; AP2, AP2 domain protein; ARF, auxin response factor. Click here for file

Additional file 5 **The stemloop primers and qRT-PCR primers for the 10 selected miRNAs.** Detailed information of stem loop primers and qRT-PCR primers. Click here for file

## References

[B1] Hymowitz T On the domestication of the soybean Economic Botany 1970 24 408 421

[B2] Ge Y Zhu Y Lv D Dong T Wang W Tan S Liu C Zou P Research on responses of wild soybean to alkaline stress Pratacultural Science 2009 26 47 52

[B3] Ge Y Li Y Zhu Y Bai X Lv D Guo D Ji W Cai H Global transcriptome profiling of wild soybean roots under NaHCO3 treatment BMC Plant Biol 2010 10 153 20653984 10.1186/1471-2229-10-153 PMC3017823

[B4] Aladdin H Xu D Conserved salt tolerance quantitative trait locus (QTL) in wild and cultivated soybeans Breeding Science 2008 58 355 359

[B5] Tuyen D Lal S Xu D Identification of a major QTL allele from wild soybean (Glycihe soja Sieb. & Zucc.) for increasing alkaline salt tolerance in soybean Theor Appl Genet 2010 121 229 236 20204319 10.1007/s00122-010-1304-y

[B6] Hyten D Song Q Zhu Y Choi I Nelson R Costa J Specht J Shoemaker R Cregan P Impacts of genetic bottlenecks on soybean genome diversity Proc Natl Acad Sci USA 2006 103 16666 16671 17068128 10.1073/pnas.0604379103 PMC1624862

[B7] Lam HM Xu X Liu X Chen W Yang G Wong FL Li MW He W Qin N Wang B Resequencing of 31 wild and cultivated soybean genomes identifies patterns of genetic diversity and selection Nat Genet 2010 42 12 1053 1059 21076406 10.1038/ng.715

[B8] Kim MY Lee S Van K Kim TH Jeong SC Choi IY Kim DS Lee YS Park D Ma J Whole-genome sequencing and intensive analysis of the undomesticated soybean (Glycine soja Sieb. and Zucc.) genome Proc Natl Acad Sci USA 2010 107 51 22032 22037 21131573 10.1073/pnas.1009526107 PMC3009785

[B9] Kochian LV Hoekenga OA Pineros MA How do crop plants tolerate acid soils? Mechanisms of aluminum tolerance and phosphorous efficiency Annu Rev Plant Biol 2004 55 459 493 15377228 10.1146/annurev.arplant.55.031903.141655

[B10] Ma JF Syndrome of aluminum toxicity and diversity of aluminum resistance in higher plants International Review of Cytolog 2007 264 225 252 10.1016/S0074-7696(07)64005-4 17964924

[B11] Kochian L Piñeros M Hoekenga O The physiology, genetics and molecular biology of plant aluminum resistance and toxicity Plant Soil 2005 274 1 175

[B12] Delhaize E Ryan PR Hebb DM Yamamoto Y Sasaki T Matsumoto H Engineering high-level aluminum tolerance in barley with the ALMT1 gene Proc Natl Acad Sci USA 2004 101 42 15249 15254 15471989 10.1073/pnas.0406258101 PMC524075

[B13] Huang CF Yamaji N Mitani N Yano M Nagamura Y Ma JF A bacterial-type ABC transporter is involved in aluminum tolerance in rice Plant Cell 2009 21 2 655 667 19244140 10.1105/tpc.108.064543 PMC2660611

[B14] Liu J Magalhaes JV Shaff J Kochian LV Aluminum-activated citrate and malate transporters from the MATE and ALMT families function independently to confer Arabidopsis aluminum tolerance Plant J 2009 57 3 389 399 18826429 10.1111/j.1365-313X.2008.03696.x

[B15] Huang CF Yamaji N Chen Z Ma JF A tonoplast-localized half-size ABC transporter is required for internal detoxification of aluminum in rice Plant J 2012 69 5 857 867 22035218 10.1111/j.1365-313X.2011.04837.x

[B16] Sasaki T Yamamoto Y Ezaki B Katsuhara M Ahn S Ryan P Delhaize E E MH: A wheat gene encoding an aluminum-activated malate transporter Plant J 2004 37 645 653 14871306 10.1111/j.1365-313x.2003.01991.x

[B17] Iuchi S Koyama H Iuchi A Kobayashi Y Kitabayashi S Kobayashi Y Ikka T Hirayama T Shinozaki K Kobayashi M Zinc finger protein STOP1 is critical for proton tolerance in Arabidopsis and coregulates a key gene in aluminum tolerance Proc Natl Acad Sci USA 2007 104 23 9900 9905 17535918 10.1073/pnas.0700117104 PMC1887543

[B18] Yamaji N Huang CF Nagao S Yano M Sato Y Nagamura Y Ma JF A zinc finger transcription factor ART1 regulates multiple genes implicated in aluminum tolerance in rice Plant Cell 2009 21 10 3339 3349 19880795 10.1105/tpc.109.070771 PMC2782276

[B19] Bianchi-Hall CM Thomas E Carter J Bailey M Al E Aluminum tolerance associated with quantitative trait loci derived from soybean PI 416937 in hydroponics Crop Science 2000 40 538 545

[B20] Nian H Yang Z Huang H Yan X Matsumoto H Citrate secretion induced by aluminum stress may not be a key mechanism responsible for differential aluminum tolerance of some soybean genotypes Journal of Plant Nutrition 2004 27 2047 2066

[B21] Ermolayev V Weschke W Manteuffel R Comparison of Al-induced gene expression in sensitive and tolerant soybean cultivars J Exp Bot 2003 54 2745 2756 14623943 10.1093/jxb/erg302

[B22] Ragland M Soliman K Two genes induced by Al in soybean roots Plant Physiol 1997 114 3959159957

[B23] Duressa D Soliman K Chen D Identification of aluminum responsive genes in Al-tolerant soybean line PI 416937 International Journal of Plant Genomics 2010 2010 1 13 10.1155/2010/164862 PMC2952814 20953355

[B24] Brodersen P Sakvarelidze-Achard L Bruun-Rasmussen M Dunoyer P Yamamoto YY Sieburth L Voinnet O Widespread translational inhibition by plant miRNAs and siRNAs Science 2008 320 1185 1190 18483398 10.1126/science.1159151

[B25] Bartel DP MicroRNAs: target recognition and regulatory functions Cell 2009 136 215 233 19167326 10.1016/j.cell.2009.01.002 PMC3794896

[B26] Jones-Rhoades MW Bartel DP Bartel B MicroRNAS and their regulatory roles in plants Annu Rev Plant Biol 2006 57 19 53 16669754 10.1146/annurev.arplant.57.032905.105218

[B27] Mallory AC Vaucheret H Functions of microRNAs and related small RNAs in plants Nat Genet 2006 38 S31 S36 16736022 10.1038/ng1791

[B28] Ruiz-Ferrer V Voinnet O Roles of plant small RNAs in biotic stress responses Annu Rev Plant Biol 2009 60 485 510 19519217 10.1146/annurev.arplant.043008.092111

[B29] Fahlgren N Howell MD Kasschau KD Chapman EJ Sullivan CM Cumbie JS Givan SA Law TF Grant SR Dangl JL High-throughput sequencing of Arabidopsis microRNAs: evidence for frequent birth and death of MIRNA genes PLoS One 2007 2 2 e219 17299599 10.1371/journal.pone.0000219 PMC1790633

[B30] Sunkar R Zhou X Zheng Y Zhang W Zhu J Identification of novel and candidate miRNAs in rice by high throughput sequencing BMC Plant Biol 2008 8 25 18312648 10.1186/1471-2229-8-25 PMC2292181

[B31] Zhao CZ Xia H Frazier TP Yao YY Bi YP Li AQ Li MJ Li CS Zhang BH Wang XJ Deep sequencing identifies novel and conserved microRNAs in peanuts (Arachis hypogaea L.) BMC Plant Biol 2010 10 3 20047695 10.1186/1471-2229-10-3 PMC2826338

[B32] Chi X Yang Q Chen X Wang J Pan L Chen M Yang Z He Y Liang X Yu S Identification and characterization of microRNAs from peanut (Arachis hypogaea L.) by high-throughput sequencing PLoS One 2011 6 11 e27530 22110666 10.1371/journal.pone.0027530 PMC3217988

[B33] Li Y Zhang Q Zhang J Wu L Qi Y Zhou JM Identification of microRNAs involved in pathogen-associated molecular pattern-triggered plant innate immunity Plant Physiol 2010 152 4 2222 2231 20164210 10.1104/pp.109.151803 PMC2850012

[B34] Chen L Zhang Y Ren Y Xu J Zhang Z Wang Y Genome-wide identification of cold-responsive and new microRNAs in Populus tomentosa by high-throughput sequencing Biochemical and Biophysical Research Communication 2011 417 897 896 10.1016/j.bbrc.2011.12.070 22209794

[B35] Wang T Chen L Zhao M Tian Q Zhang W Identification of drought-responsive microRNAs in Medicago truncatula by genome-wide high-throughput sequencing BMC Genomics 2011 12 367 21762498 10.1186/1471-2164-12-367 PMC3160423

[B36] Zhou ZS Zeng HQ Liu ZP Yang ZM Genome-wide identification of Medicago truncatula microRNAs and their targets reveals their differential regulation by heavy metal Plant Cell Environ 2012 35 1 86 99 21895696 10.1111/j.1365-3040.2011.02418.x

[B37] Li H Dong Y Yin H Wang N Yang J Liu X Wang Y Wu J Li X Characterization of the stress associated microRNAs in Glycine max by deep sequencing BMC Plant Biol 2011 11 170 22112171 10.1186/1471-2229-11-170 PMC3267681

[B38] Song QX Liu YF Hu XY Zhang WK Ma B Chen SY Zhang JS Identification of miRNAs and their target genes in developing soybean seeds by deep sequencing BMC Plant Biol 2011 11 5 21219599 10.1186/1471-2229-11-5 PMC3023735

[B39] Subramanian S Fu Y Sunkar R Barbazuk WB Zhu JK Yu O Novel and nodulation-regulated microRNAs in soybean roots BMC Genomics 2008 9 160 18402695 10.1186/1471-2164-9-160 PMC2335117

[B40] Wang Y Li P Cao X Wang X Zhang A Li X Identification and expression analysis of miRNAs from nitrogen-fixing soybean nodules Biochemical and Biophysical Research Communication 2009 378 4 799 803 10.1016/j.bbrc.2008.11.140 19084500

[B41] Wong CE Zhao YT Wang XJ Croft L Wang ZH Haerizadeh F Mattick JS Singh MB Carroll BJ Bhalla PL MicroRNAs in the shoot apical meristem of soybean Journal of Experimental Botony 2011 62 8 2495 2506 10.1093/jxb/erq437 21504877

[B42] Yong-xin L Wei C Ying-peng H Quan Z Mao-zu G Wen-bin L In silico detection of novel MicroRNAs genes in soybean genome Agricultural Sciences in China 2011 10 1336 1345

[B43] Zhang B Pan X Stellwag EJ Identification of soybean microRNAs and their targets Planta 2008 229 1 161 182 18815805 10.1007/s00425-008-0818-x

[B44] Jing W Chun-yan L Li-wei Z Jia-lin W Guo-hua H Jun-jie D Qing-shan C MicroRNAs Involved in the Pathogenesis of Phytophthora Root Rot of Soybean (Glycine max) Agricultural Sciences in China 2011 10 1159 1167

[B45] Kulcheski FR de Oliveira LF Molina LG Almerao MP Rodrigues FA Marcolino J Barbosa JF Stolf-Moreira R Nepomuceno AL Marcelino-Guimaraes FC Identification of novel soybean microRNAs involved in abiotic and biotic stresses BMC Genomics 2011 12 307 21663675 10.1186/1471-2164-12-307 PMC3141666

[B46] Chen R Hu Z Zhang H Identification of microRNAs in wild soybean (Glycine soja) J Integr Plant Biol 2009 51 12 1071 1079 20021554 10.1111/j.1744-7909.2009.00887.x

[B47] Addo-Quaye C Eshoo TW Bartel DP Axtell MJ Endogenous siRNA and miRNA targets identified by sequencing of the Arabidopsis degradome Curr Biol 2008 18 10 758 762 18472421 10.1016/j.cub.2008.04.042 PMC2583427

[B48] German MA Pillay M Jeong DH Hetawal A Luo SJ Janardhanan P Kannan V Rymarquis LA Nobuta K German R Paoli ED Lu C Schroth G Meyers BC Green PJ Global identification of microRNA-target RNA pairs by parallel analysis of RNA ends Nat Biotechnol 2008 26 941 946 18542052 10.1038/nbt1417

[B49] Li YF Zheng Y Addo-Quaye C Zhang L Saini A Jagadeeswaran G Axtell MJ Zhang W Sunkar R Transcriptome-wide identification of microRNA targets in rice Plant J 2010 62 5 742 759 20202174 10.1111/j.1365-313X.2010.04187.x

[B50] Chen L Wang T Zhao M Tian Q Zhang WH Identification of aluminum-responsive microRNAs in Medicago truncatula by genome-wide high-throughput sequencing Planta 2012 235 2 375 386 21909758 10.1007/s00425-011-1514-9

[B51] Pantaleo V Szittya G Moxon S Miozzi L Moulton V Dalmay T Burgyan J Identification of grapevine microRNAs and their targets using high-throughput sequencing and degradome analysis Plant J 2010 62 6 960 976 20230504 10.1111/j.0960-7412.2010.04208.x

[B52] Martinez G Forment J Llave C Pallas V Gomez G High-throughput sequencing, characterization and detection of new and conserved cucumber miRNAs PLoS One 2011 6 5 e19523 21603611 10.1371/journal.pone.0019523 PMC3095615

[B53] Song C Wang C Zhang C Korir NK Yu H Ma Z Fang J Deep sequencing discovery of novel and conserved microRNAs in trifoliate orange (Citrus trifoliata) BMC Genomics 2010 11 431 20626894 10.1186/1471-2164-11-431 PMC2996959

[B54] The Rfam database ftp://ftp.sanger.ac.uk/pub/databases/Rfam/9.1/

[B55] The Repbase databasehttp://www.girinst.org/repbase/update/index.html

[B56] The Glysince max database ftp://ftp.jgi-psf.org/pub/JGI_data/phytozome/v7.0/Gmax/annotation/Gmax_109_transcript.fa.gz

[B57] Meyers BC Axtell MJ Bartel B Bartel DP Baulcombe D Bowman JL Cao X Carrington JC Chen X Green PJ Criteria for Annotation of Plant MicroRNAs Plant Cell 2008 20 3186 3190 19074682 10.1105/tpc.108.064311 PMC2630443

[B58] Rajagopalan R Vaucheret H Trejo J Bartel DP A diverse and evolutionarily fluid set of microRNAs in Arabidopsis thaliana Genes Dev 2006 20 24 3407 3425 17182867 10.1101/gad.1476406 PMC1698448

[B59] Ambros V Bartel B Bartel DP Burge CB Carrington JC Chen X Dreyfuss G Eddy SR Griffiths-Jones S Marshall M A uniform system for microRNA annotation RNA 2003 9 3 277 279 12592000 10.1261/rna.2183803 PMC1370393

[B60] Zuker M Mfold web server for nucleic acid folding and hybridization prediction Nucleic Acids Res 2003 31 13 3406 3415 12824337 10.1093/nar/gkg595 PMC169194

[B61] Addo-Quaye C Snyder JA Park YB Li YF Sunkar R Axtell MJ Sliced microRNA targets and precise loop-first processing of MIR319 hairpins revealed by analysis of the Physcomitrella patens degradome RNA 2009 15 12 2112 2121 19850910 10.1261/rna.1774909 PMC2779683

[B62] Du Z Zhou X Ling Y Zhang Z Su Z agriGO: a GO analysis toolkit for the agricultural community Nucleic Acids Res 2010 38 Web Server issue W64 W70 20435677 10.1093/nar/gkq310 PMC2896167

[B63] Zhou ZS Huang SQ Yang ZM Bioinformatic identification and expression analysis of new microRNAs from Medicago truncatula Biochemical and Biophysical Research Communication 2008 374 3 538 542 10.1016/j.bbrc.2008.07.083 18662674

[B64] Sabehat A Weiss D Lurie S Heat-shock proteins and cross-tolerance in plants Physiol Plant 1998 103 437 441

[B65] Ding Y Chen Z Zhu C Microarray-based analysis of cadmium-responsive microRNAs in rice (Oryza sativa) J Exp Bot 2011 62 10 3563 3573 21362738 10.1093/jxb/err046 PMC3130178

[B66] Guo HS Xie Q Fei JF Chua NH MicroRNA directs mRNA cleavage of the transcription factor NAC1 to down-regulate auxin signals for Arabidopsis lateral root development Plant Cell 2005 17 5 1376 1386 15829603 10.1105/tpc.105.030841 PMC1091761

[B67] Zhao B Liang R Ge L Li W Xiao H Lin H Ruan K Jin Y Identification of drought-induced microRNAs in rice Biochemical and Biophysical Research Communication 2007 354 2 585 590 10.1016/j.bbrc.2007.01.022 17254555

[B68] Liu HH Tian X Li YJ Wu CA Zheng CC Microarray-based analysis of stress-regulated microRNAs in Arabidopsis thaliana RNA 2008 14 5 836 843 18356539 10.1261/rna.895308 PMC2327369

[B69] Zhao B Ge L Liang R Li W Ruan K Lin H Jin Y Members of miR-169 family are induced by high salinity and transiently inhibit the NF-YA transcription factor BMC Mol Biol 2009 10 29 19351418 10.1186/1471-2199-10-29 PMC2670843

[B70] Zhou L Liu Y Liu Z Kong D Duan M Luo L Genome-wide identification and analysis of drought-responsive microRNAs in Oryza sativa J Exp Bot 2010 61 4157 4168 20729483 10.1093/jxb/erq237

[B71] Li WX Oono Y Zhu J He XJ Wu JM Iida K Lu XY Cui X Jin H Zhu JK The Arabidopsis NFYA5 transcription factor is regulated transcriptionally and post-transcriptionally to promote drought resistance Plant Cell 2008 20 8 2238 2251 18682547 10.1105/tpc.108.059444 PMC2553615

[B72] Zhang W Gao S Zhou X Chellappan P Chen Z Zhou X Zhang X Fromuth N Coutino G Coffey M Bacteria-responsive microRNAs regulate plant innate immunity by modulating plant hormone networks Plant Mol Biol 2011 75 1–2 93 105 21153682 10.1007/s11103-010-9710-8 PMC3005105

[B73] Radwan O Liu Y Clough SJ Transcriptional analysis of soybean root response to Fusarium virguliforme, the causal agent of sudden death syndrome Molecula Plant-Microbe Interactions 2011 24 8 958 972 10.1094/MPMI-11-10-0271 21751852

[B74] Rhoades MW Reinhart BJ Lim LP Burge CB Bartel B Bartel DP Prediction of plant microRNA targets Cell 2002 110 4 513 520 12202040 10.1016/s0092-8674(02)00863-2

[B75] Jones-Rhoades MW Bartel DP Computational identification of plant microRNAs and their targets, including a stress-induced miRNA Mol Cell 2004 14 6 787 799 15200956 10.1016/j.molcel.2004.05.027

[B76] Sunkar R Girke T Jain PK Zhu JK Cloning and characterization of microRNAs from rice Plant Cell 2005 17 5 1397 1411 15805478 10.1105/tpc.105.031682 PMC1091763

[B77] Buhtz A Springer F Chappell L Baulcombe DC Kehr J Identification and characterization of small RNAs from the phloem of Brassica napus Plant J 2008 53 5 739 749 18005229 10.1111/j.1365-313X.2007.03368.x

[B78] Kurihara Y Kaminuma E Matsui A Kawashima M Tanaka M Morosawa T Ishida J Mochizuki Y Shinozaki K Toyoda T Transcriptome Analyses Revealed Diverse Expression Changes in ago1 and hyl1 Arabidopsis Mutants Plant and Cell Phydiology 2009 50 1715 1720 10.1093/pcp/pcp109 19633021

[B79] Erik A Van Der B Jonathan DGJ The NB-ARC domain: a novel signalling motif shared by plant resistance gene products and regulators of cell death in animals Curr Biol 1998 8 226 667 10.1016/s0960-9822(98)70145-9 9545207

[B80] Swiderski MR Doris B Jonathan DGJ The TIR domain of TIR-NB-LRR resistance proteins is a signaling domain involved in cell death induction Molecular Plant-Microbe Interations 2009 22 157 165 10.1094/MPMI-22-2-0157 19132868

[B81] Tang P Zhang Y Sun X Tian C Yang S Ding J Disease resistance signature of the leucine-rich repeat receptor-like kinase genes in four plant species Plant Sci 2010 179 399 406

[B82] Kollmeier M Felle HH Horst WJ Genotypical differences in aluminum resistance of maize are expressed in the distal part of the transition zone. Is reduced basipetal auxin flow involved in inhibition of root elongation by aluminum? Plant Physiol 2000 122 3 945 956 10712559 10.1104/pp.122.3.945 PMC58931

[B83] Sun P Tian QY Chen J Zhang WH Aluminium-induced inhibition of root elongation in Arabidopsis is mediated by ethylene and auxin Journal of Experimental Botony 2010 61 2 347 356 10.1093/jxb/erp306 PMC2803203 19858117

[B84] Wang JW Wang LJ Mao YB Cai WJ Xue HW Chen XY Control of root cap formation by MicroRNA-targeted auxin response factors in Arabidopsis Plant Cell 2005 17 8 2204 2216 16006581 10.1105/tpc.105.033076 PMC1182483

[B85] Xie Q Frugis G Colgan D Chua NH Arabidopsis NAC1 transduces auxin signal downstream of TIR1 to promote lateral root development Genes Dev 2000 14 23 3024 3036 11114891 10.1101/gad.852200 PMC317103

[B86] Duval M Hsieh TF Kim SY Thomas TL Molecular characterization of AtNAM: a member of the Arabidopsis NAC domain superfamily Plant Mol Biol 2002 50 2 237 248 12175016 10.1023/a:1016028530943

[B87] Hou N You J Pang J Xu M Chen G Yang Z The accumulation and transport of abscisic acid in soybean (Glycine max L.) under aluminum stress Plant Soil 2010 330 127 137

[B88] Reyes JL Chua N ABA induction of miR159 controls transcript levels of two MYB factors during Arabidopsis seed germination Plant J 2007 49 592 606 17217461 10.1111/j.1365-313X.2006.02980.x

[B89] Mittler R Oxidative stress, antioxidants and stress tolerance Trends Plant Sci 2002 7 405 410 12234732 10.1016/s1360-1385(02)02312-9

[B90] Beffagna N Buffoli B Busi C Modulation of reactive oxygen species production during osmotic stress in Arabidopsis thaliana cultured cells: involvement of the plasma membrane Ca2+-ATPase and H+-ATPase Plant Cell Physiol 2005 46 8 1326 1339 15937326 10.1093/pcp/pci142

[B91] Shabala S Baekgaard L Shabala L Fuglsang AT Cuin TA Nemchinov LG Palmgren MG Endomembrane Ca2+-ATPases play a significant role in virus-induced adaptation to oxidative stress Plant Signal Behav 2011 6 7 1053 1056 21633195 10.4161/psb.6.7.15634 PMC3257794

[B92] ESTs and GSSs of Glycine soja registered in ncbi http://www.ncbi.nlm.nih.gov/Taxonomy/Browser/wwwtax.cgi?id=3848

