# Supplementary figures and images for: Identification of wild soybean miRNAs and their target genes responsive to aluminum stress
Source: BMC Plant Biol. 2012 Oct 5;12:182. doi: 10.1186/1471-2229-12-182 (PMC3519564; doi:10.1186/1471-2229-12-182)

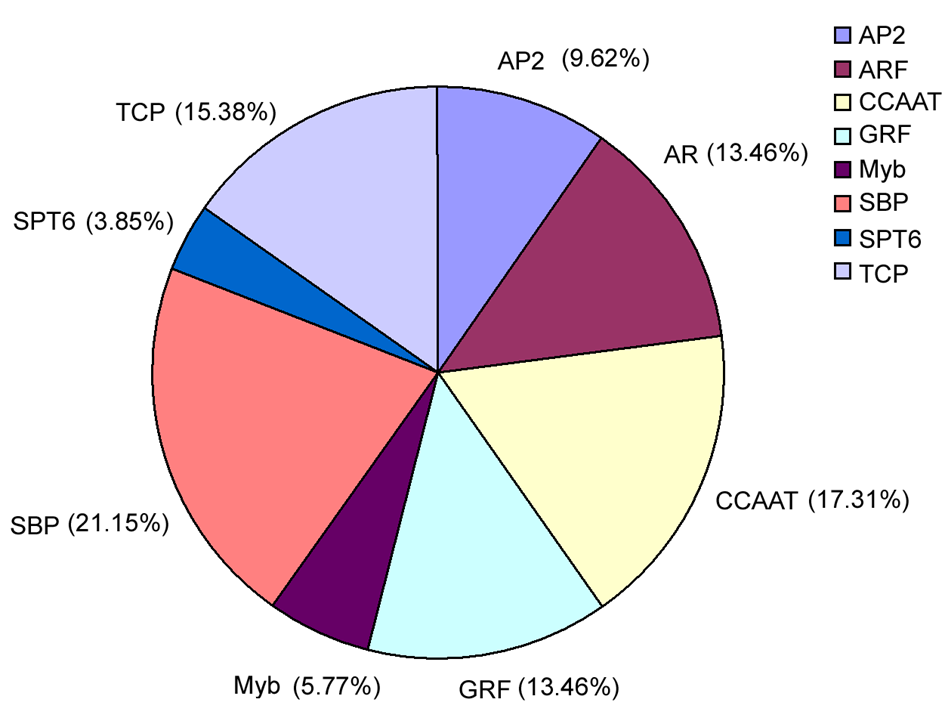

Supplement: Additional file 4 — The distribution of the transcription regulators cleaved by known miRNAs. The percentage indicates proportion of the different transcription regulators out of the total number of transcription regulators for the known miRNAs. CCAAT, CCAAT-binding transcription factor; Myb, Myb family of transcription factors; SBP, SBP domain proteins; TCP, TCP family of transcription factors; SPT6, transcription elongation factor SPT6; WRC, growth regulating factor; AP2, AP2 domain protein; ARF, auxin response factor. [file 1471-2229-12-182-S4.tiff]
